# Apolipoprotein ε4 polymorphism does not modify the association between body mass index and high-density lipoprotein cholesterol: a cross-sectional cohort study

**DOI:** 10.1186/1476-511X-10-167

**Published:** 2011-09-23

**Authors:** Catherine R Rahilly-Tierney, Donna K Arnett, Kari E North, James S Pankow, Steven C Hunt, R Curtis Ellison, J Michael Gaziano, Luc Djoussé

**Affiliations:** 1 Massachusetts Veterans Epidemiology and Information Research Center (MAVERIC), Boston VA Healthcare, Boston, Massachusetts, USA; 2 Geriatric Research Education and Clinical Centers (GRECC), Boston VA Healthcare, Boston, Massachusetts, USA; 3 Division of Aging, Brigham & Women's Hospital, Boston, Massachusetts, USA; 4 Harvard Medical School, Boston, Massachusetts, USA; 5 Department of Epidemiology, University of Alabama at Birmingham School of Public Health, Birmingham, Alabama, USA; 6 Carolina Center for Genome Science, University of North Carolina Gillings School of Global Public Heatlh, Chapel Hill, North Carolina, USA; 7 Division of Epidemiology & Community Health, University of Minnesota School of Public Health, Minneapolis, Minnesota, USA; 8 Cardiovascular Genetics Division, University of Utah, Salt Lake City, Utah, USA; 9 Section of Preventive Medicine & Epidemiology, Boston University School of Medicine, Boston, Massachusetts, USA

**Keywords:** HDL cholesterol, body mass index, genetic epidemiology, apolipoproteins, lipid metabolism, adiposity

## Abstract

**Background:**

We sought to examine whether ε4 carrier status modifies the relation between body mass index (BMI) and HDL. The National Heart, Lung, and Blood Institute Family Heart Study included 657 families with high family risk scores for coronary heart disease and 588 randomly selected families of probands in the Framingham, Atherosclerosis Risk in Communities, and Utah Family Health Tree studies. We selected 1402 subjects who had ε4 carrier status available. We used generalized estimating equations to examine the interaction between BMI and ε4 allele carrier status on HDL after adjusting for age, gender, smoking, alcohol intake, mono- and poly-unsaturated fat intake, exercise, comorbidities, LDL, and family cluster.

**Results:**

The mean (standard deviation) age of included subjects was 56.4(11.0) years and 47% were male. Adjusted means of HDL for normal, overweight, and obese BMI categories were 51.2(± 0.97), 45.0(± 0.75), and 41.6(± 0.93), respectively, among 397 ε4 carriers (p for trend < 0.0001) and 53.6(± 0.62), 51.3(± 0.49), and 45.0(± 0.62), respectively, among 1005 non-carriers of the ε4 allele (p-value for trend < 0.0001). There was no evidence for an interaction between BMI and ε4 status on HDL(p-value 0.39).

**Conclusion:**

Our findings do not support an interaction between ε4 allele status and BMI on HDL.

## Introduction

HDL cholesterol (HDL) has been associated with lower risk of coronary heart disease (CHD) in observational and trial cohorts [1-8]. Apolipoprotein E (APOE) is a protein component of triglyceride-rich lipoproteins and thus participates in the transport of lipids among various cells in the body [9]. There are 3 major isoforms of apolipoprotein E in modern populations, encoded by alleles ε2, ε3, and ε4. The ε4 allele has been associated with lower levels of HDL and higher risk of CHD events [10]. Higher body mass index (BMI) has also been found to be associated with lower HDL levels and increased risk of CHD [11-13]. In studies including subjects at average risk for CHD, previous researchers have not found that a BMI-genotype interaction impacts HDL levels [14-16]. However, to the best of our knowledge, no researchers have reported whether APOE ε4 allele carrier status modifies the relation between higher BMI and lower HDL levels in a cohort enriched with subjects and higher-than-average risk for CHD. This question may be important from a clinical standpoint, to determine whether carriers of Apo ε4 are at any disadvantage when attempting to lower their BMI to achieve a more favorable lipid profile. Therefore, to evaluate whether there is a significant interaction between BMI and APOE ε4 carrier status on HDL levels, we examined the relationship between these in a cohort of 1402 men and women selected from the National Heart, Lung, and Blood Institute Family Heart Study (NHLBI FHS).

## Methods

### Population

The NHLBI FHS is a multicenter, population-based study that includes probands recruited from three parent studies: the Framingham Heart Study (Framingham, MA), the Atherosclerosis Risk in Communities Study (Forsythe County, NC and Minneapolis, MN), and the Utah Family Health Tree (Salt Lake City, UT). Between 1993 and 1995, 2,000 randomly selected participants and 2,000 with family histories of CHD enrolled in the 3 parent studies were mailed invitations to provide information regarding the health of themselves, their parents, their children, and their siblings. Of these, 3,150 returned responses, and family members of these responders were contacted. From the 22,908 persons who provided information, 588 families were randomly selected and an additional 657 with high family risk scores were also selected. Family risk score was calculated by comparing the actual incidence of CHD to the age- and sex-adjusted expected incidence of CHD within a family. Enrollees underwent study examinations during which height, weight and blood pressure were documented, and 12-hour fasting blood samples were taken on which lipids and other parameters were measured, and from which DNA was stored. Genotyping for the APOE allele was performed on a selection of FHS participants. More detailed description of the methods of the NHLBI FHS have been described previously [17]. The FHS is supported by the National Heart, Lung, and Blood Institute (NHLBI) (grant numbers U01 HL56563, U01 HL56564, U01 HL56565, U01 HL56566, U01 HL56567, U01 HL56568, U01 HL56569, and K01-HL70444). Each participant in the FHS gave informed consent and the study protocol was reviewed and approved by the respective Internal Review Boards at each of the participating institutions.

For this study, we selected subjects with information on APOE genotype and with complete data on lipids, lifestyle factors, and co-morbidities. Due to small numbers of non-white subjects, participants of other races (54 subjects) were excluded from this study, as were 34 subjects who were using cholesterol modifying therapy. Finally 37 subjects were excluded with the ε2/ε4 genotype, as the ε2 allele has been associated with higher levels of HDL while ε4 carrier status has been associated with lower HDL levels [18].

### Exposure

APOE genotyping was performed using polymerase chain reaction (PCR) to amplify a 267-base pair fragment from exon 3 of the APOE gene. The PCR product was digested using the *Hha-1 *restriction endonuclease, which resulted in a specific banding pattern for the 3 isoforms of the apolipoprotein E protein when separated by polyacrylamide gel electrophoresis and silver stained [19]. Subjects with genotypes of ε3/ε4 or ε4/ε4 were carriers; subjects with genotypes of ε2/ε2, ε2/ε3, or ε3/ε3 were non-carriers.

Height and weight documented during the study examination were used to calculate body mass index (BMI). BMI was treated both as a continuous and a categorized variable (BMI < 25 kg/m^2^, 25 ≤ BMI < 30 kg/m^2^, and BMI ≥ 30 kg/m^2^) in the analyses described below.

### Lipid measurements

During the study visits, a fasting blood sample was collected in evacuated tubes without additives, spun at 3000xg for 10 minutes at 4°C, and stored at -70° until they were processed. HDL, total cholesterol, and triglyceride concentrations were measured using a COBAS FARA high-speed centrifugal analyzer (Roche Diagnostic Systems, Montclair, New Jersey).

### Covariates

Information on lifestyle factors including smoking (currently smoking versus not currently smoking), alcohol intake (consumption of 2 or more beverages daily), and physical activity (minutes of exercise per day) was obtained by interview during the study visit. Mono- and poly-unsaturated fat intake (MUFA and PUFA, in grams) was determined using a previously validated food frequency questionnaire administered by study staff during the study interview and a nutrient database from Harvard University supplemented by manufacturer's information [20]. Participants provided self-reported information on co-morbities including angina, CHD, stroke, cancers, and hypertension. A subject was deemed to have diabetes mellitus if he was taking hypoglycemic agents, if a physician had told the subject that he had diabetes mellitus, or if fasting blood glucose was ≥ 126 mg/dL. In subjects with triglycerides < 400 mg/dL, LDL (LDL) was calculated using measured HDL, total cholesterol, and triglycerides. LDL was measured directly on EDTA plasma using ultracentrifugation on EDTA plasma in subjects with triglycerides ≥ 400 mg/dL.

### Statistical Analyses

The chi-square test was used to determine whether the APOE allele distribution was in Hardy-Weinberg equilibrium in this population prior to exclusions. We compared characteristics of the cohort by ε4 carrier status and by BMI category, using t-tests to compare continuous variables and Cochran-Mantel-Haenszel tests to compare categorical variables. We then used generalized estimating equations (GEE, using the LSMEANS option in PROC GENMOD in SAS) to determine adjusted means and standard errors for HDL across BMI categories, in subgroups by ε4 carrier status. GEE adjusts for familial clustering in the NHLBI FHS. Means were adjusted for age, categorized BMI, gender, current smoking status, alcohol consumption (≥ 2 beverages daily versus non or < 2 daily), minutes of exercise daily, mono- and PUFA intake in grams, angina, stroke, hypertension, coronary heart disease, diabetes mellitus, and LDL. We determined the parameter estimate and p-value for a categorized BMIxε4 carrier product term in a model including the main effects of categorized BMI and carrier status, plus age, gender, lifestyle factors, co-morbidities, and LDL. In secondary analyses, we examined whether a continuous BMIxε4 carrier product term was significantly associated with HDL after adjusting for the main effects of continuous BMI and carrier status and age, then additionally adjusting for gender, lifestyle factors, co-morbidities, and LDL. We did not include triglycerides in adjusted models due to collinearity between the outcome (HDL) and triglycerides as previously modeled by others [21].

In a sensitivity analysis, we examined whether there was a significant association between continuous BMIxε4 carrier product term and HDL in a sub-cohort consisting of one individual (randomly selected) per family, and in subgroups of participants defined by CHD risk (members of randomly selected families versus members of families with increased CHD risk). To examine whether there was a significant interaction between BMI and APOE genotype (in contrast to carrier status for the ε4 allele) we treated genotype as an ordinal variable (ε2/ε2, ε2/ε3, ε3/ε3, ε3/ε4, ε4/ε4) and included a continuous BMIxgenotype product term in the model. To examine whether a BMIxε4 carrier status interaction was significantly associated with HDL in subgroups by gender, we repeated the analysis including the main effects of BMI and ε4 carrier status and their interaction term in subgroups of men and women. All analyses were performed using SAS version 9.2 (SAS Institute Incorporated, Cary, North Carolina).

## Results

A total of 1402 NHLBI FHS participants from 662 families were included in our study. Each family contributed an average of 2 participants to our cohort (range, 1-11 family members). The mean (standard deviation, SD) age of the study cohort was 56.4 (11.0) years, 47% were male, and about 75% had a BMI within the overweight or obese range (≥ 25 kg/m^2^). As expected given the selection method for the study, there was a high prevalence of smoking (13%), angina (14%), CHD (18%), and hypertension (34%). The distribution of the APOE alleles was in Hardy-Weinberg equilibrium (p-value 0.91). Overall, 28% of subjects were carriers of the APOE ε4 allele. Table 1 presents characteristics of the cohort by ε4 carrier status. The mean HDL among ε4 carriers was significantly lower and the mean triglycerides significantly higher than those among non-carriers. Carriers of the ε4 allele were younger and more likely than non-carriers to be hypertensive. Table 2 presents characteristics of the cohort according to BMI category. Subjects with higher BMI were more likely to be male, have a higher fat intake, exercised for fewer minutes daily, had lower HDL, and were more likely to have diabetes mellitus or hypertension.

**Table 1:** Characteristics of n = 1402 National Heart, Lung, and Blood, Institute Family Heart Study participants, by apolipoprotein ε4 carrier status.

	Carriers of ε4	Non-carriers of ε4	p-value
Characteristic	n = 397 (28.3%)	n = 1005 (71.7%)	
Age, mean (SD)*	54.9(10.9)	57.0(11.0)	0.001
Male, no. (%)*	193(48.6)	472(47.0)	0.58
BMI, kg/m^2^, mean (SD)*	28.3(5.3)	28.4(5.6)	0.89
Current smoker, no. (%)	45(11.3)	138(13.7)	0.23
≥ 2 alcoholic drinks daily, no. (%)	197(49.6)	497 (49.5)	0.95
Monounsaturated fat intake, g/day, mean (SD)	24.7(13.9)	23.3(12.5)	0.06
Polyunsaturated fat intake, g/day, mean (SD)	9.1(5.1)	8.7(4.6)	0.17
Exercise in minutes/day, mean (SD)	26.8(37.3)	28.2(34.7)	0.51
HDL, mg/dL, mean (SD)*	45.8(14.1)	49.1(15.8)	0.0003
LDL, mg/dL, mean (SD)	132.8(37.0)	128.8(35.5)	0.06
Triglycerides, mg/dL, mean (SD)	178.7(102.0)	164.4(109.3)	0.03
Diabetes mellitus, no. (%)	27(6.8)	95(9.5)	0.11
Hypertension, no. (%)	116(29.2)	364(36.2)	0.01
Coronary heart disease, no. (%)	72(18.1)	176(17.5)	0.78
Angina, no. (%)	46(11.6)	150(14.9)	0.10
Stroke, no. (%)	13(3.3)	35(3.5)	0.85
Cancer, no. (%)	45(11.3)	111(11.0)	0.88

*BMI = Body Mass Index; HDL = high-density lipoprotein cholesterol; kg/m^2 ^= kilograms/meters-squared; LDL = low-density lipoprotein cholesterol; mg/dL = milligrams/deciliter; No. = number; SD = standard deviation

**Table 2:** Characteristics of the n = 1402 participants, by category of body mass index.

	BMI ≤ 25 kg/m^2^*	25 < BMI ≤ 30 kg/m^2^	BMI > 30 kg/m^2^	
Characteristic	n = 407 (29.0%)	n = 565 (40.3%)	n = 430 (30.7%)	p-value
Age, mean (SD)*	56.2 (11.1)	57.1 (11.3)	55.8 (10.6)	0.57
Carrier ε4 allele, no. (%)*	112 (27.5)	162 (28.7)	123 (28.6)	0.73
Male, no. (%)	143 (35.1)	330 (58.4)	192 (44.7)	0.008
BMI, kg/m^2^, mean (SD)*	22.8 (1.7)	27.4 (1.4)	34.9 (4.6)	--
Current smoker, no. (%)	69 (17.0)	77 (13.6)	37 (8.6)	0.0003
≥ 2 alcoholic drinks daily, no. (%)	209 (51.4)	287 (50.8)	198 (46.1)	0.12
Monounsaturated fat intake, mean (SD)	21.8 (12.6)	24.0 (12.5)	25.0 (37.0)	0.0004
Polyunsaturated fat intake, mean (SD)	8.4 (4.4)	8.9 (4.7)	9.2 (5.1)	0.02
Exercise in minutes/day, mean (SD)	30.1 (33.8)	28.6 (35.3)	24.5 (37.0)	0.02
HDL, mg/dL, mean (SD)*	55.1 (17.5)	46.5 (14.2)	44.0 (12.4)	< 0.0001
LDL, mg/dL, mean (SD)*	129.7 (35.1)	131.5 (38.9)	128.2 (32.5)	0.53
Triglycerides, mg/dL, mean (SD)	134.0 (105.7)	179.5 (103.0)	186.6 (107.5)	< 0.0001
Diabetes mellitus, no. (%)	17 (4.2)	53 (9.4)	52 (12.1)	< 0.0001
Hypertension, no. (%)	103 (25.3)	202 (35.8)	175 (40.7)	< 0.0001
Coronary heart disease, no. (%)	57 (14.0)	127 (22.5)	64 (14.9)	0.79
Angina, no. (%)	36 (8.9)	107 (18.9)	53 (12.3)	0.17
Stroke, no. (%)	9 (2.2)	28 (5.0)	11 (2.6)	0.82
Cancer, no. (%)	38 (9.3)	73 (12.9)	45 (10.5)	0.63

*BMI = Body Mass Index; HDL = high-density lipoprotein cholesterol; kg/m^2 ^= kilograms/meters-squared; LDL = low-density lipoprotein cholesterol; mg/dL = milligrams/deciliter; No. = number; SD = standard deviation

Table 3 presents multivariate adjusted means ± standard errors of HDL in each BMI category, in subgroups by ε4 carrier status. As BMI increases, HDL decreases in both ε4 carriers and non-carriers (p-value for trend < 0.0001 in each subgroup). In an analysis including the main effects of categorized BMI and ε4 carrier status, there was no significant association between a categorized BMIxε4 carrier product term and HDL (p-value 0.75, results not shown). Table 3 also presents the parameter estimates and p-values for BMI as a continuous variable and for a continuous BMI × ε4 carrier product term. The association between continuous BMI and HDL remains robust, with or without adjustment for the BMI × ε4 carrier product term and other co-variates including gender, lifestyle factors, LDL, and co-morbidities. In age- and multivariate-adjusted parameter estimates, there was no significant association between the continuous BMI × ε4 carrier product term and HDL (multivariate parameter estimate 0.11, p-value 0.39).

**Table 3:** Adjusted mean (± standard error, SE) high-density lipoprotein cholesterol in each category of body mass index(BMI), by ε4 carrier status (top section) and parameter estimates ± SE and p-values for continuous BMI and continuous BMI × ε4 carrier status (bottom section).

	n	ε4 Carriers	n	ε4 Non-carriers	Overall cohort	
HDL^a^, mg/dL, mean ± SE*					Parameter estimate(SE)	p-value
BMI ≤ 25 kg/m^2^*	112	51.2 ± 0.97	295	53.6 ± 0.62		
25 < BMI ≤ 30 kg/m^2^	162	45.0 ± 0.75	403	51.3 ± 0.49		
BMI ≥ 30 kg/m^2^	123	41.6 ± 0.93	307	45.0 ± 0.62		
P-value for trend		< 0.0001		< 0.0001		

BMI						
Adjusted for age and carrier status					-0.69(0.07)	< 0.0001
Adjusted for age, carrier status, and interaction					-0.70(0.08)	< 0.0001
Multivariate-adjusted^b^					-0.75(0.07)	< 0.0001
Continuous BMI × carrier term						
Adjusted for age, BMI, and carrier status					0.02(0.14)	0.90
Multivariate-adjusted^b^					0.10(0.12)	0.39

*BMI = Body Mass Index; HDL = high-density lipoprotein cholesterol; mg/dL = milligrams/deciliter; kg/m^2 ^= kilograms/meters-squared

^┼^Multivariate-adjusted estimates for HDL are adjusted for age, gender, categorized BMI, current smoker (yes/no), alcohol intake(≥ 2 beverages daily, yes/no), monounsaturated fat intake in grams, polyunsaturated fat intake in grams, minutes of exercise daily, angina, coronary heart disease, stroke, cancer, hypertension, diabetes mellitus, and low-density lipoprotein cholesterol

^b^Multivariate-adjusted parameter estimates are adjusted for age, gender, continuous BMI, ε4 carrier status, continuous BMIxε4 carrier interaction, current smoker (yes/no), alcohol intake (≥ 2 beverages daily, yes/no), monounsaturated fat intake in grams, polyunsaturated fat intake in grams, minutes of exercise daily, angina, coronary heart disease, stroke, cancer, hypertension, diabetes mellitus, and LDL.

In a sensitivity analysis including one member per family, results again revealed that after adjusting for the main effects of BMI and ε4 carrier status, a continuous BMI × carrier product term remained non-significant (results not shown). Repeating the analysis in subgroups consisting of participants from randomly selected families versus those with increased CHD risk, we found that results were similar (not shown). In an analysis treating APOE genotype as an ordinal variable and including a continuous BMI × genotype product term, the interaction between genotype and BMI remained non -significant (p-value for interaction 0.55; p-value for main effects of continuous BMI and APOE genotype were 0.0008 and 0.24 respectively, when interaction term included in the model, results not shown). In an analysis including the main effects of continuous BMI and ε4 carrier status and a continuous BMI × carrier product term in subgroups of men and women, results were unchanged (results not shown).

## Discussion

In a cohort of 1402 men and women, we found that APOE ε4 carrier status did not modify the strong association between higher BMI and lower HDL levels. We found that increasing BMI was significantly associated with decreasing HDL, regardless of whether or not subjects were carriers of the ε4 allele. These findings were robust in subgroups by gender and in a cohort in which only one member per family was included. In analyses examining whether there was interaction between APOE genotype and the BMI-HDL association, we again found a strong association between increasing BMI and decreasing HDL levels, regardless of genotype.

In subjects at average risk for CHD, previous researchers have found that APOE genotype does not modify the association between measures of adiposity, including BMI and waist-to-hip ratio, and HDL levels. In a Canadian cohort including 1788 men and women of varying CHD risk, Lussier-Cacan et al found that the relationship between BMI and HDL did not vary significantly across APOE genotypes [14]. We similarly found no difference in the relation between BMI and HDL in ε4 carriers compared to non-carriers. In a cohort of 759 young adults (aged 20 to 32 years) followed longitudinally, obese individuals had much higher prevalence of low HDL levels compared to non-obese individuals, but this did not differ across APOE genotype categories [15]. Our study demonstrates that the lack of modification of the BMI-HDL relation by ε4 carrier status also extends to populations enriched with subjects at higher risk for CHD.

APOE is synthesized by the liver as part of VLDL particles. In cholesterol transport, it serves as a ligand for several processes that cycle triglycerides from VLDL into HDL subtypes. The ε4 isoform of APOE is less effective in binding to lipoprotein receptors than the ε2 or ε3 isoforms, so in individuals with APOE ε4 the pathway between triglyceride-rich VLDL particles and small, denser, more easily metabolized HDL particles is less effective [9]. Though further research would be necessary to support any speculation regarding the metabolic pathway between BMI, APOE, and HDL, our findings may suggest that in the triglyceride-rich milieu associated with adiposity, the effectiveness of the APOE as a ligand becomes immaterial as the pathway between VLDL and HDL subtypes is overwhelmed. In a supplemental analysis, we examined whether an interaction between BMI and APOE genotype impacted HDL levels and found no significant association. This lends further support to the idea that the effectiveness of one player, APOE, in the complex pathway that metabolizes lipids becomes less important in the context of high levels of substrate entering that pathway, as is the case in subjects with high BMI.

There are several advantages to using the NHLBI FHS database to examine whether there is a significant interaction between BMI and ε4 carrier status in influencing HDL levels, including detailed information on several lifestyle factors that are associated with HDL (alcohol intake, smoking, diet, and exercise). Misclassification of the main exposure variable, namely BMI, is unlikely as height and weight were determined by study staff during the study visit. However there are some limitations to our study. The small number of non-Caucasian subjects disallowed us from examining whether there is a significant BMIxgenotype association with HDL in persons of other racial or ethnic groups. We may have been under-powered to detect a small effect of a BMI × APOE interaction on HDL levels. Confirming that no significant interaction exists in larger cohorts remains an important area of future research.

We found that ε4 carrier status does not modify the well-established association between increasing BMI and decreasing HDL levels. Our findings suggest that carriers of the ε4 allele who lower their BMI in an attempt to improve their lipid profile may not be at a disadvantage when compared to non-carriers of the allele. However, further study in larger cohorts and with subjects of other races or ethnicity are warranted to definitively establish whether ε4 carrier status modifies the BMI-HDL association.

## List of Abbreviations

APOE: apolipoprotein E; BMI: body mass index; CI: confidence interval; CHD: coronary heart disease; GEE: generalized estimating equations; HR: hazard ratio; HDL: high density lipoprotein cholesterol; LDL: low density lipoprotein cholesterol; MUFA: mono-unsaturated fatty acid; NHLBI FHS: National Heart Lung & Blood Institute Family Heart Study; PUFA: poly-unsaturated fatty acids; SD: standard deviation; VLDL: very low density lipoprotein cholesterol

## Competing interests

The authors declare that they have no competing interests.

## Authors' contributions

CRT and LD planned the study and designed the analysis. LD acquired the data. CRT executed the statistical analysis and prepared the tables. LD and CRT interpreted the results, and CRT drafted the manuscript. DKA, KEN, JSP, SCH, and JMG were instrumental in editing the manuscript to its present form. All authors have read and approve the final manuscript.
